# Progesterone elevation on the day of human chorionic gonadotropin administration adversely affects the outcome of IVF with transferred embryos at different developmental stages

**DOI:** 10.1186/s12958-015-0075-3

**Published:** 2015-08-04

**Authors:** Yan Huang, En-yin Wang, Qing-yun Du, Yu-jing Xiong, Xiao-yi Guo, Yi-ping Yu, Ying-pu Sun

**Affiliations:** Department of Reproductive Medical Center, First Affiliated Hospital of Zhengzhou University, Jianshe Dong Road, Erqi District, Zhengzhou City, Henan Province People’s Republic of China

**Keywords:** IVF, Progesterone, Pregnancy rate, GnRH agonist, Blastocyst

## Abstract

**Background:**

The effect of progesterone elevation (PE) on the day of human chorionic gonadotropin (hCG) administration on the pregnancy outcomes of in vitro fertilization/intracytoplasmic sperm injection (IVF/ICSI) cycles is a matter of ongoing debate. The replacement of cleavage-stage embryos with blastocyst-stage embryos for transfer was proposed to avoid the possible impairment of PE in fresh cycles. This study aimed to assess the association between PE on the day of human chorionic gonadotropin (hCG) administration and clinical pregnancy rates (CPRs) in IVF/ICSI cycles with embryos transferred at different developmental stages (cleavage and blastocyst). Moreover, a secondary aim was to determine the thresholds at which PE has a detrimental effect on CPRs.

**Methods:**

This single-center retrospective cohort study included more than 10,000 patients undergoing day 3 cleavage-stage embryo transfer (ET) and 1146 patients undergoing day 5 blastocyst-stage embryo transfer (ET) using gonadotropin and GnRH agonist for controlled ovarian stimulation.

**Results:**

Serum PE was inversely associated with CPRs in both cleavage- and blastocyst-stage ET cycles. In the day 3 ET cycles, CPRs (progesterone levels < 0.5 ng/ml, 49.2 %) significantly declined when the progesterone concentration reached 1.0 ng/ml (45.5 %) and decreased further when the progesterone concentration increased to 1.5 ng/ml (36.2 %). In the day 5 blastocyst-stage ET cycles, patients with serum progesterone levels ≥1.75 ng/ml had significantly lower CPRs (31.3 % VS. 41.4 %, *p* < 0.001) compared to patients with serum progesterone levels <1.75 ng/ml. The negative association of PE with CPRs was noted in both ET groups, even after adjusting for confounders. Furthermore, the developmental stage of the transferred embryos was not linked to the effect of PE on CPRs because the interaction between the developmental stage of the transferred embryos and PE was not significant.

**Conclusions:**

PE on the day of hCG administration is associated with decreased CPRs in GnRH agonist IVF/intracytoplasmic sperm injection (ICSI) cycles regardless of the developmental stage of the transferred embryos (cleavage versus blastocyst stage).

## Background

Despite the widespread use of gonadotropin-releasing hormone (GnRH) analogues for pituitary down-regulation, progesterone elevation (PE), which refers to an increase in serum progesterone concentrations, still occurs at different frequencies on the day of human chorionic gonadotropin (hCG) administration for final oocyte maturation in fresh in vitro fertilization (IVF) cycles [1]. Moreover, the question of whether PE on the day of hCG administration affects the outcomes of IVF is still being debated [2–12]. Some studies have indicated that PE does not affect the probability of pregnancy in IVF [2–4, 12]; however, other studies have concluded that PE resulted in a decreased probability of pregnancy [5–11]. The use of simple bivariate analyses and arbitrary cut-off levels in most of these studies may explain these varying results. Recently, a large-sample meta-analysis confirmed the adverse effect of PE on IVF pregnancy outcomes [1].

The precise endocrinological mechanism underlying the adverse effects of PE is still unclear; however, oocyte quality [13] and endometrial receptivity [14, 15] may play roles in this process. The hypothesis regarding endometrial receptivity is seemingly more convincing because of the results of gene expression studies on the endometrium [16, 17] and studies showing that the live birth rates of women implanted with frozen-thawed embryos or donor oocytes from fresh cycles did not significantly differ between those with PE and those without PE [1, 18].

Several strategies have been proposed to avoid the possible detrimental effects of PE in fresh IVF cycles [19, 20]. One strategy is to replace cleavage-stage embryos with blastocyst-stage embryos prior to transfer because evidence suggests that PE does not decrease pregnancy rates in day 5 single blastocyst transfer cycles [7, 21]. However, the reliability of these results has not been confirmed, and some researchers have found that PE can still impair IVF outcomes, even in blastocyst transfers [8, 13, 22].

Hence, the aim of this study was to assess the association between PE on the day of hCG administration and the clinical pregnancy rates (CPRs) of GnRH agonist IVF/intracytoplasmic sperm injection (ICSI) cycles with the transfer of embryos at different developmental stages. Moreover, a secondary aim was to determine the thresholds at which PE has a detrimental effect on CPRs.

## Material and methods

### Study population

This was a single-center retrospective cohort study. Patients who were undergoing IVF with gonadotropin and a GnRH agonist for controlled ovarian stimulation were enrolled from January 2010 to October 2014. A total of 10,864 patients were assigned to the day 3 embryo transfer (ET) group undergoing cleavage-stage ET, and 1146 patients were assigned to the day 5 ET group undergoing blastocyst-stage ET. The Ethics Committee of the First Hospital of Zhengzhou University approved the research protocol for this study.

The exclusion criteria were as follows: (i) individuals in the first or second oocyte or sperm donation cycles and (ii) couples in which either member had chromosomal abnormalities. Each patient was included in the study only once, during either the first or second IVF/ICSI cycle.

The following patient characteristics were evaluated: age, basal follicle-stimulating hormone (bFSH) level, body mass index (BMI), cause of infertility, duration of infertility and type of infertility. Other measured parameters included the dose and type of gonadotropin; the duration of gonadotropin administration; serum endometrial thickness and P/E2/LH levels (on the hCG day); and the numbers of retrieved oocytes, two pronuclear (2PN) oocytes, 2PN cleavage embryos, available embryos and transferred embryos. All data were collected from computerized databases.

### Controlled ovarian stimulation and embryo transfer

The selected protocols included a standard long GnRH agonist protocol and a prolonged or modified prolonged GnRH agonist protocol [23, 24]. Clinicians selected an appropriate protocol for each patient on a case-by-case basis based on patient characteristics.

Pituitary suppression was achieved by injecting triptorelin acetate (Decapeptyl® 0.1 mg [Ferring, Germany] or Diphereline® 0.1/3.75 mg [Ipsen, France]) until the serum levels of E2, follicle-stimulating hormone (FSH) and LH were <30 mIU/mL, <5 mIU/mL and <5 mIU/mL, respectively. Controlled ovarian stimulation was initiated with several types of gonadotropin, with follicle stimulating hormone (FSH) activity (Gonal-F® 75 IU [Serono, Switzerland], Fostiman® 75 IU [IBSA, Switzerland], or Puregon® 50 IU [N. V. Organon, Netherlands]) or with FSH combined with luteinizing hormone (LH) activity (hMG®, Livzon, China). In general, the initial dose of gonadotropins, which ranged from 112.5 IU to 400 IU, was based on the individual’s age, BMI, bFSH and response during previous stimulated cycles and the presence of polycystic ovary syndrome (PCOS).

After at least 4 days of stimulation, doses were adjusted based on the ovarian response, as evaluated using ultrasonography and serum E2 measurement. When more than three follicles reached 17 mm, hCG was injected, and after 36–37 h, oocyte retrieval was performed.

A grading criterion was used to evaluate the quality of the cleavage-stage embryos [25]. Grade 1 and Grade 2 embryos with ≥6 symmetrical blastomeres of equal size were considered top-quality embryos. Generally, patients with three or more top-quality cleavage-stage embryos were likely to be candidates for blastocyst culture. A scoring system developed by Gardner was applied for blastocyst-stage embryo grading [26]. A blastocyst with a grade ≥3BB was considered a top-quality embryo. According to national regulations, the number of embryos per patient, and other individual requirements, 1–3 of the best day 3 embryos or 1–2 of the best day 5 blastocysts were selected for transfer. In addition, when the progesterone level had increased to more than 2.5 ng/ml before hCG, all embryos were frozen and transferred in sequential frozen ET cycles.

### Serum hormone measurements

Basal FSH was measured on days 2–4 of the menstrual cycle before the IVF cycle began. Hormones, including LH, E2 and P, were assessed during controlled ovarian stimulation (once every 2–4 days at first and once a day during the late follicular phase and on the day of hCG administration in each cycle). All blood samples were taken during fasting, generally between 6:30 and 7:30 am. We used an individual immunoanalyzer (Roche Cobas e411; Roche Diagnostics, Mannheim, Germany) and the same assays for all hormone measurements during the entire study.

Serum progesterone levels were measured using an electrochemiluminescence immunoassay with Progesterone II (Cobas 12145383). The assay’s limit was 0.03 ng/ml, and its sensitivity was 0.15 ng/ml. The intra-assay and inter-assay coefficients of variation were 3.0 and 5.5 %, respectively.

### Outcome variables

The CPR was the primary outcome. The presence of an intrauterine gestational sac (with positive cardiac pulsations on ultrasound) at 35 or 45 days after ET was considered to indicate a clinical pregnancy. Other rates were also calculated: the implantation rate (IR) = number of sacs (both within and outside the uterine cavity)/the number of transferred embryos; the fertilization rate = the number of 2PN oocytes/the number of oocytes retrieved; and the cleavage rate = the number of 2PN cleavage-stage embryos/the number of 2PN oocytes.

### Statistical analysis

The continuous variables are described using means and standard deviations (SDs) or medians and interquartile ranges (IQRs). The categorical variables are described as percentages of the total. Student’s *t*-test or the Mann–Whitney *U* test were applied for single comparisons, and one-way analysis of variance (ANOVA) followed by the least significant difference (LSD) *t* test or the Kruskal-Wallis (KW) test followed by all pairwise multiple comparisons were used for multiple comparisons of the continuous variables based on the normality of the distribution. For categorical variables, the chi-square test or Fisher's exact test was performed.

Because the relationship between serum progesterone levels and pregnancy rates was assumed to be non-linear [19], the patients in the day 3 and day 5 ET groups were divided into the following eight distinct groups for analyses according to their serum progesterone levels on the day of hCG: <0.50, 0.50-074, 0.75-0.99, 1.00-1.24, 1.25-1.49, 1.50-1.74, 1.75-1.99 and ≥2.00 ng/ml [5, 6]. To assess the association between PE and CPRs and determine the thresholds at which PE has a detrimental effect on CPRs, CPRs were calculated for each group and subjected to a Mantel-Haenszel test for trend analysis. Furthermore, the odds ratios (ORs) and 95 % confidence intervals (CIs) of the CPRs were calculated to confirm the thresholds by comparing each group to the group with the lowest progesterone level (<0.50 ng/ml). Pairwise comparisons were also made between adjacent groups, as follows: <0.50 and 0.50-0.74 ng/ml, 0.50-0.74 and 0.75-0.99 ng/ml, 0.75-0.99 and 1.00-1.24 ng/ml, 1.00-1.24 and 1.25-1.49 ng/ml, 1.25-1.49 and 1.50-1.74 ng/ml, 1.50-1.74 and 1.75-1.99 ng/ml, and 1.75-1.99 and ≥2.00 ng/ml. Moreover, the developmental stage of the transferred embryos (cleavage versus blastocyst stage), PE (defined as an unordered categorical variable according to progesterone levels: <0.50, 0.50-074, 0.75-0.99, 1.00-1.24, 1.25-1.49, 1.50-1.74, 1.75-1.99 and ≥2.00 ng/ml) and the interaction terms between them were examined in multivariate logistic regression analyses and evaluated together with other confounders to assess the effect of the developmental stage of the transferred embryos on the association PE with CPR. The confounders included female age, BMI, gonadotropin dose, the number of retrieved oocytes, the number of transferred embryos, the number of top-quality transferred embryos, and serum E2 levels on the day of hCG administration [27, 28].

After appropriate progesterone threshold levels were determined for the day 3 and day 5 ET groups, the serum progesterone levels were subdivided based on the threshold and were subjected to multivariate logistic regression analyses while controlling for other confounders to more accurately determine the effect of serum PE on CPRs. Additionally, the patients in these two groups were classified into several subgroups according to each group’s progesterone cut-off level. The demographic and clinical characteristics of the individuals in these subgroups were then compared.

## Results

### Patient characteristics

The included patients’ baseline characteristics are shown in Table 1. A total of 10,864 patients underwent day 3 ET, and a total of 1146 patients underwent day 5 ET in IVF/ICSI/IVF + ICSI cycles. The median (IQR) bFSH levels of these patients were 7.16 (2.5) and 6.57 (2.0) IU/L, respectively. A total of 84.3 % patients in the day 3 ET group and 88.1 % patients in the day 5 ET group were in their first IVF/ICSI cycles. Several relatively clear causes of infertility were present in the patients in the day 3 and day 5 ET groups, including tubal pathology (44.5 and 58.5 %, respectively), male factors (27.5 and 29.8 %, respectively), advanced age (≥35 years, 11.1 and 14.6 %, respectively), PCOS (6.2 and 11.5 %, respectively), and endometriosis (3.5 and 3.3 %, respectively).

**Table 1:** Patients’ baseline characteristics

Parameter	Day 3 ET	Day 5 ET
	(*n* = 10,864)	(*n* = 1146)
Age (years)	31.3 ± 5.1	29.7 ± 4.5
BMI (kg/m^2^)	22.0 (3.0)	22.0 (4.5)
bFSH (IU/L)	7.16 (2.5)	6.57 (2.0)
Duration of infertility (years)	3 (4)	3 (3)
Primary infertility (%)	49.2	55.2
Second infertility (%)	50.8	44.8
First treatment cycle (%)	84.3	88.1
Cause of infertility (%)		
tubal pathology	44.5	58.5
male factors	27.5	29.8
advanced age (≥35 years)	11.1	14.6
PCOS	6.2	11.5
endometriosis	3.5	3.3
pelvic pathology	6.5	6.5
others	3.8	4.1
unexplained factors	13.3	11.2
ART (%)		
IVF	69.8	72.2
ICSI	28.8	25.6
IVF + ICSI	1.4	2.2
Stimulation protocol (%)		
GnRH-a long protocol	82.8	93.5
GnRH-a prolonged (modified) protocol	11.1	5.8

*Note:* ART: assisted reproductive technology; IVF + ICSI: IVF + rescue ICSI or IVF + late ICSI; GnRH-a: gonadotropin-releasing hormone agonist; PCOS: polycystic ovary syndrome

Table 2 presents the clinical, oocyte and embryo parameters from controlled ovarian stimulation to ET. The median (IQR) duration and dose of gonadotropin were 11.0 (2.0) days and 2100 (1275) IU, respectively, in the day 3 group and 11.0 (2.0) days and 1637.5 (787) IU, respectively, in the day 5 group. The mean (±SD) level of serum progesterone, the E2 level on the day of hCG, and the median (IQR) number of retrieved oocytes were 0.83 ± 0.44 ng/ml, 4421.8 ± 2570 pg/ml and 10 (7), respectively, in the day 3 group; these values were much higher in the day 5 group (1.03 ± 0.43 ng/ml, 7696.2 ± 2659.7 pg/ml and 18 (5), respectively). Double ET occurred in most of the individuals in the day 3 ET group (87 %), and single blastocyst-stage ET occurred in most of the individuals in the day 5 ET group (91.4 %).

**Table 2:** Clinical/oocyte/embryo parameters from ovarian stimulation to embryo transfer

Parameter	Day 3 ET	Day 5 ET
	(*n* = 10,864)	(*n* = 1146)
Duration of stimulation (days)	11.0 (2.0)	11.0 (2.0)
Total dose of Gn (IU)	2100 (1275)	1637.5 (787)
Serum progesterone (ng/ml), hCG day	0.83 ± 0.44	0.99 ± 0.43
Serum E2 (pg/ml), hCG day	4421.8 ± 2570.0	7696.2 ± 2659.7
Serum LH (IU/L), hCG day	1.88 (1.13)	1.46 (0.87)
Endometrial thickness (mm), hCG day	12.0 (1.8)	12.0(1.3)
Oocytes retrieved (*n*)	10 (7)	18 (5)
2PN oocytes (*n*)	6 (5)	14 (4)
2PN cleavage embryos (*n*)	6 (5)	13 (4)
Available embryos (*n*)	5 (4)	9 (4)
ET with a single embryo (%)	1.3	91.4
ET with two embryos (%)	87	8.6
ET with more than two embryos (%)	11.7	/

*Note*: Values are the mean ± SD or median (IQR) unless otherwise noted; hCG: human chorionic gonadotropin; Gn: gonadotropin; E2: estrogen; LH: luteinizing hormone; ET: embryo transfer

### The relationship between serum progesterone levels and clinical pregnancy rates

**Fig. 1:**
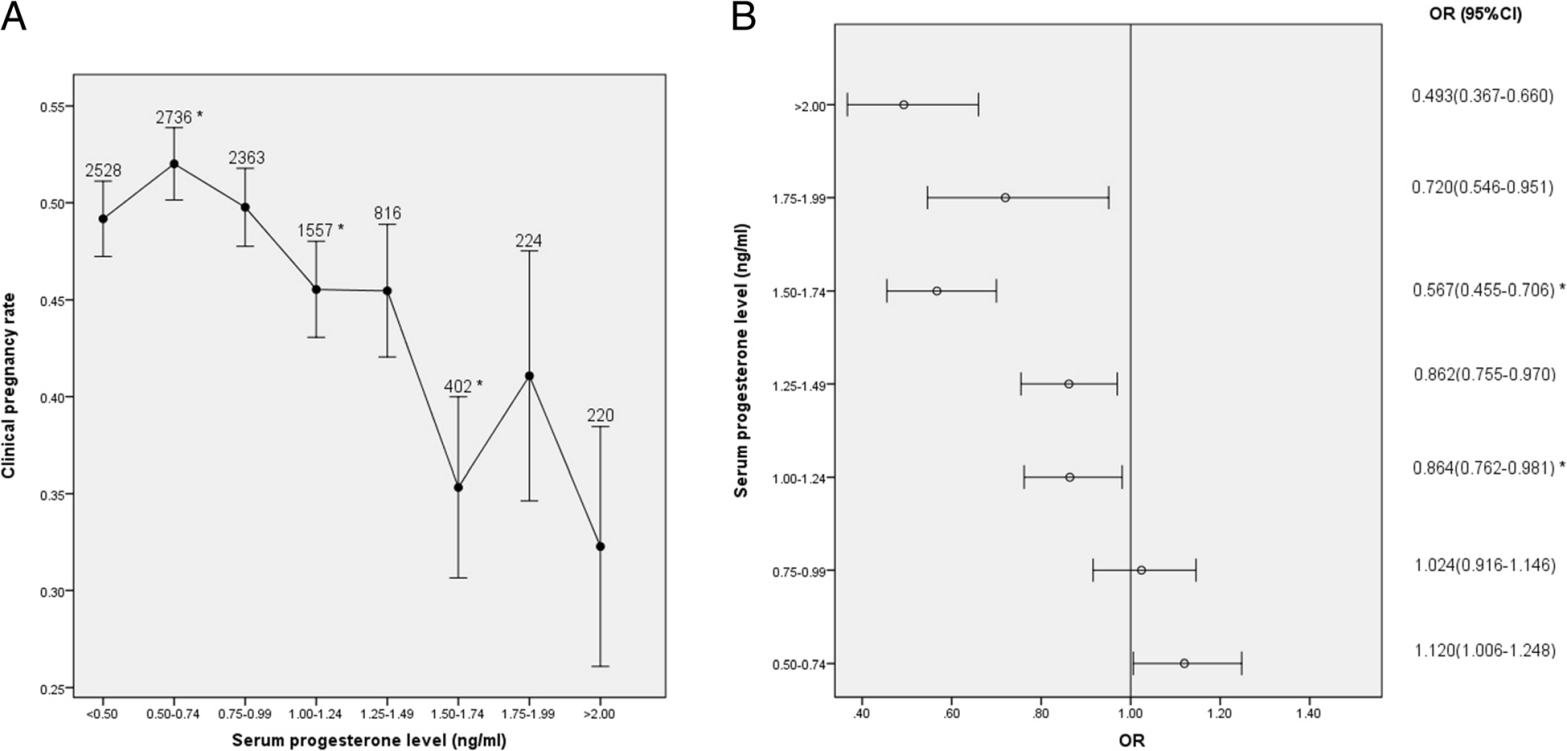
Relationship between serum progesterone levels and CPRs in the day 3 ET group (**a**) Relationship between serum progesterone levels and CPRs, (**b**) CPRs according to serum progesterone levels. *p < 0.05 for comparison with the previous progesterone group; data are expressed as the OR (95 % CI) for each serum progesterone group compared with the group with the lowest progesterone level (<0.5 ng/ml); OR: odds ratio; CI: confidence interval

The relationships between serum progesterone levels on the day of hCG administration and CPRs in the day 3 ET group are shown in Fig. 1.

Serum progesterone levels were inversely related to CPRs in the day 3 ET group, as shown in Fig. 1a (*p* < 0.0001 for overall trend). A decrease in CPRs was observed when the serum progesterone level was ≥1.0 ng/ml, and an additional reduction was observed when the serum progesterone level was ≥1.5 ng/ml. By comparing each group to the group with the lowest progesterone level (<0.5 ng/ml), the ORs (95 % CIs) of CPRs were calculated (Fig. 1b). The CPRs of these five groups with progesterone levels ≥1.0 ng/ml were significantly lower than the CPRs of the group with the lowest progesterone level (<0.5 ng/ml). Furthermore, when the 0.75-0.99 ng/ml group was compared with the 1.00-1.24 ng/ml group (49.8 % VS. 45.5 %, *p* = 0.009) and the 1.25-1.49 ng/ml group was compared with the 1.50-1.74 ng/ml group (45.5 % VS. 35.3 %, *p* = 0.002), the differences were statistically significant. These data suggest that progesterone began to have a detrimental effect on the probability of pregnancy in day 3 cleavage-stage ET cycles once the serum progesterone level reached 1.0 ng/ml. When the progesterone concentration reached 1.5 ng/ml, the detrimental effect appeared to worsen.

**Fig. 2:**
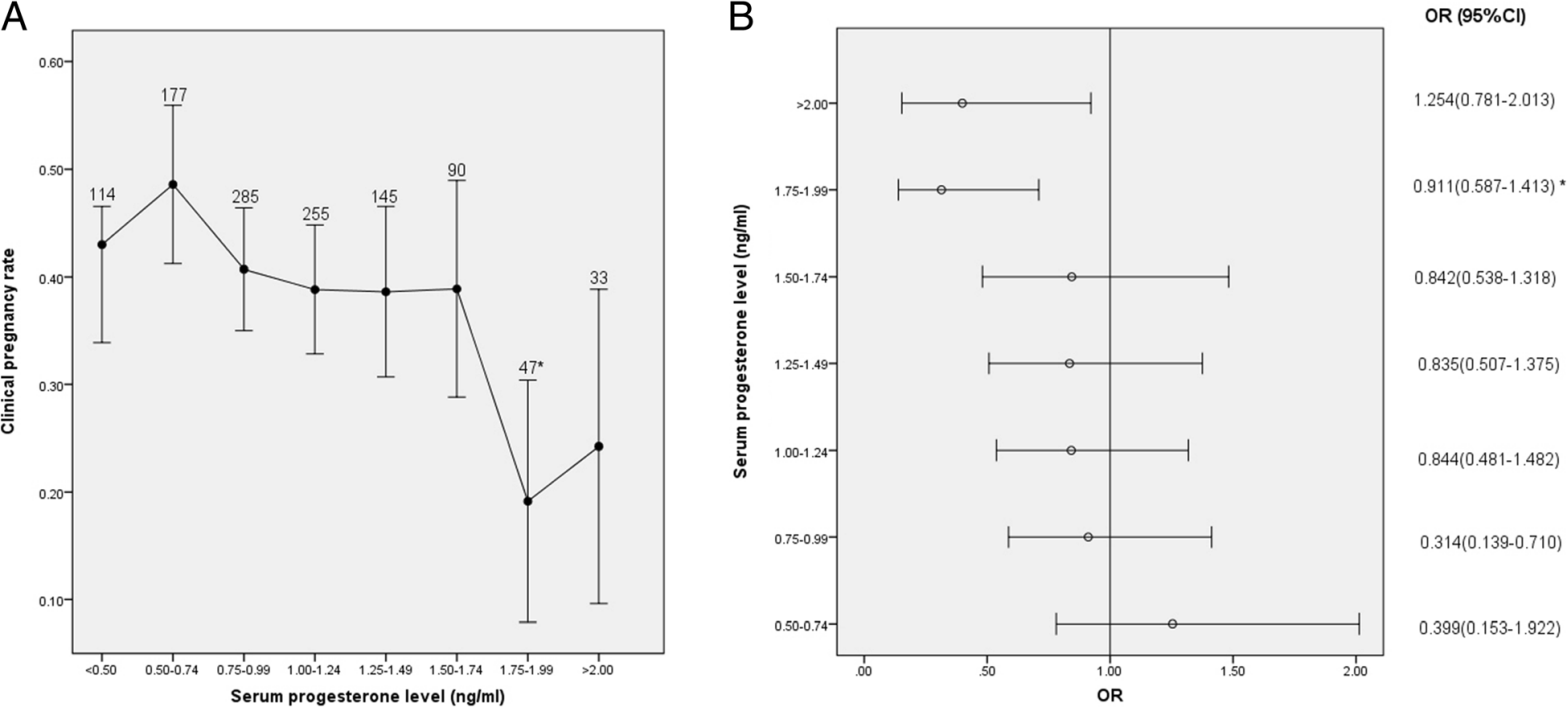
Relationship between serum progesterone levels and CPRs in the day 5 ET group (**a**) Relationship between serum progesterone levels and CPRs, (**b**) CPRs according to serum progesterone levels. *p < 0.05 for comparison with the previous progesterone group; data are expressed as the OR (95 % CI) for each serum progesterone group compared with the group with the lowest progesterone level (<0.5 ng/ml); OR: odds ratio; CI: confidence interval

The relationships between serum progesterone levels and CPRs in the day 5 ET group are shown in Fig. 2.

The patients’ serum progesterone levels were inversely associated with CPRs, as shown in Fig. 2a (*p* = 0.0076 for the overall trend). CPRs decreased with gradually higher levels of serum progesterone, especially when the serum progesterone level was ≥1.75 ng/ml. The ORs (95 % CI) of CPRs for each of the groups compared with the group with the lowest progesterone level (<0.5 ng/ml) are shown in Fig. 2b. Statistically significant reductions in CPRs were only observed in the 1.75-1.99 (OR: 0.307 [0.146-0.646], *p* = 0.001) and ≥2 ng/ml groups (OR: 0.414 [0.184-0.934], *p* = 0.029). Moreover, when adjacent groups were compared, the CPRs differed significantly only between the 1.5-1.74 and 1.75-1.99 ng/ml groups (38.9 % VS. 19.1 %, *p* = 0.019 and 42.6 % VS. 22.2 %, *p* = 0.021, respectively). These results imply that a serum progesterone concentration of 1.75 ng/ml was likely to be the cut-off level at which progesterone has an adverse impact on clinical pregnancy in IVF cycles with day 5 blastocyst-stage ETs.

### Multivariate analysis of factors associated with clinical pregnancy

A multivariate logistic regression analysis showed that several factors were significantly associated with CPRs (Table 3). In the day 3 ET group, serum PE was negatively associated with CPRs (OR: 0.874 [0.823-0.929], *p* < 0.001), whereas the number of top-quality transferred embryos was positively associated with CPRs (OR: 1.073 [1.001-1.150], *p = 0.047*). The results were similar for the day 5 ET group (*p* < 0.05 for each comparison); however, female age was negatively associated with CPRs (OR: 0.770 [0.606-0.980]*, p* = 0.03).

**Table 3:** Multivariate analysis of factors related to clinical pregnancy

Factors	Day 3 ET		Day 5 ET	
	OR (95 % CI)	*p*-value	OR (95 % CI)	*p*-value
Female age(years)	/	/	0.770 (0.606-0.980)	0.034
PE^a^, hCG day	0.874 (0.823-0.929)	<0.001	0.419 (0.237-0.742)	0.003
No. of transferred TQEs	1.073 (1.001-1.150)	0.047	1.331 (1.045-1.696)	0.021

*Note:* TQE: top-quality embryo; ^a^three levels of PE were defined in the day 3 ET group: <1 ng/ml, 1–1.49 ng/ml, and ≥1.5 ng/ml; two levels of PE were defined in the day 5 ET group: <1.75 ng/ml and ≥1.75 ng/ml. OR: odds ratio; CI: confidence interval

### Interaction between the developmental stage of the transferred embryos and the effect of PE on CPRs

To assess whether PE’s effect on clinical pregnancy was influenced by the developmental stage of the transferred embryos, an interaction term of developmental stage of transferred embryos and PE was included in the multivariate analyses (Table 4). Female age, PE, the developmental stage of the transferred embryos and the number of transferred top-quality embryos were all significantly related to the CPR. However, the interaction term (developmental stage of transferred embryos*PE) was not statistically significant (*p >* 0.05 for all progesterone levels), which also could not support the possible influence of the transferred embryos on PE’ effect on CPRs

**Table 4:** Interaction between the developmental stage of the transferred embryos and the effect of PE on CPRs

Factors	OR (95 % CI)	*p*-value
Female age (years)	0.930(0.872-0.993)	0.031
No. of transferred TQE	1.102(1.031-1.179)	0.004
Developmental stage	0.842(0.718-0.987)	0.004
Progesterone elevation (ng/ml)ª		<0.001
Developmental stage^b^ PE (ng/ml)		
0-0.49		0.369
0.50-0.74		0.099
0.75-0.99		0.921
1.00-1.24		0.828
1.25-1.49		0.861
1.50-1.74		0.799
1.75-1.99		0.058
2-		0.228

*Note*: TQE: top-quality embryo; OR: odds ratio; CI: confidence interval. developmental stage: cleavage versus blastocyst stage, PEª was defined as an unordered categorical variable according to progesterone levels: <0.50, 0.50-074, 0.75-0.99, 1.00-1.24, 1.25-1.49, 1.50-1.74, 1.75-1.99 and ≥2.00 ng/ml. The 95 % CIs of developmental stage ^b^PE were all covered 1

### Results of comparisons between subgroups

Demographic and clinical characteristics were compared among the subgroups within the day 3 ET group (Table 5) and the day 5 ET group (Table 6).

**Table 5:** Comparisons of demographic and clinical characteristics between subgroups of the day 3 ET group

Parameter^a^	Serum P levels (ng/ml), day 3 ET^b^		
	<1.0	1.0-1.49	≥1.5
No. of fresh cycles	7627	2373	846
Female age (years)	31.1 ± 5.1	31.8 ± 5.0*	32.1 ± 5.1
BMI (kg/m^2^)	22.7 ± 3.2	22.3 ± 3.0*	22.1 ± 3.0
Basal FSH (IU/L)	7.7 ± 2.9	7.3 ± 2.4*	7.2 ± 2.2
Total dose of Gn (IU)	2201.2 ± 940.7	2268.6 ± 879.3*	2357.1 ± 890.1*
Serum E2 (pg/ml), hCG day	3941.8 ± 2118.6	5321.0 ± 2353.8*	6201.8 ± 4598.0*
Serum LH (IU/L), hCG day	2.0 ± 1.3	1.9 ± 1.4	1.72 ± 1.9*
Endometrial thickness (mm), hCG day	12.3 ± 2.4	12.2 ± 2.5	12.0 ± 2.4*
No. of oocytes retrieved	9.3 ± 4.4	11.1 ± 4.6*	12.0 ± 4.8*
No. of 2PN oocytes	6.4 ± 3.3	7.5 ± 3.5*	7.8 ± 4.8
No. of 2PN cleavage embryos	6.4 ± 3.3	7.5 ± 3.5*	7.8 ± 4.8
No. of available embryos	5.0 ± 2.8	5.7 ± 3.0*	5.9 ± 3.2
Fertilization rate (%)	69	67.4*	65.1*
Cleavage rate (%)	97.8	97.7	97.3**
No. of transferred embryos	2.10 ± 0.34	2.12 ± 0.34*	2.14 ± 0.39
No. of transferred top-quality embryos	1.97 ± 0.55	2.00 ± 0.53**	2.02 ± 0.56*
Implantation rate (%)	36.3	31.6*	26*
CPR (%)	50.4	45.5*	36.2*

*Note:* Day 3 ET subgroups according progesterone cut-off levels: <1 ng/ml, 1–1.49 ng/ml, and ≥1.5 ng/ml. P = progesterone

^a^All values are presented as the mean ± SD or count (%)

^b^Differences between subgroups were determined using ANOVA followed by the LSD-*t* test or the Kruskal-Wallis (KW) test and subsequent pairwise multiple comparisons for continuous variables and the chi-square test or Fisher’s exact test for categorical variables

*p < 0.01 compared with the previous subgroup

**p < 0.05 compared with the previous subgroup

**Table 6:** Comparison of demographic and clinical characteristics between subgroups of the day 5 ET group

Parameter^a^	Serum P levels (ng/ml), day 5 ET		*P*-value*
	<1.75	≥1.75	
No. of fresh cycles	1066	80	
Female age (years)	29.5 ± 4.4	31.5 ± 5.2	0.002
BMI (kg/m^2^)	22.60 ± 3.31	21.95 ± 2.59	0.036
Basal FSH (IU/L)	6.61 ± 1.57	6.53 ± 1.83	NS
Total dose of Gn (IU)	1760.0 ± 635.5	2114.1 ± 680.7	<0.001
Serum E2 (pg/ml), hCG day	7583.3 ± 2595.8	9200.6 ± 3038.1	<0.001
Serum LH (IU/L), hCG day	1.6 ± 0.7	1.5 ± 0.7	NS
Endometrial thickness (mm), hCG day	12.2 ± 2.3	12.1 ± 2.5	NS
No. of oocytes retrieved	17.9 ± 5.0	19.7 ± 4.9	0.003
No. of 2PN oocytes	13.1 ± 3.8	14.4 ± 4.3	0.014
No. of 2PN cleavage embryos	12.9 ± 3.8	14.2 ± 4.3	0.003
No. of available embryos	9.7 ± 3.8	9.9 ± 4.8	0.031
Fertilization rate (%)	74.4	73.5	NS
Cleavage rate (%)	98.5	99.1	NS
No. of transferred embryos	1.08 ± 0.27	1.19 ± 0.39	0.017
ET with top-quality blastocyst (%)	58.7	52.7	NS
Implantation rate (%)	46.2	29.5	0.002
CPR (%)	41.4	21.3	<0.001

*Note:* Day 5 ET subgroups according progesterone cut-off levels: <1.75 ng/ml, ≥1.75 ng/ml; NS = not statistically significant

^a^All values are presented as the mean ± SD or count (%)

*Differences between subgroups were determined using Student’s *t*-test or the Mann–Whitney *U* test for continuous variables and the chi-square test or Fisher’s exact test for categorical variables

In the day 3 ET group (Table 5), BMI and bFSH levels were lower in the <1 ng/ml subgroup than in the ≥1.0 ng/ml subgroups, whereas the dose of gonadotropin; the numbers of 2PN oocytes, cleavage-stage embryos and available embryos; and female age were higher. The serum LH levels, endometrial thickness on the day of hCG administration and cleavage rates were lower in the ≥1.5 ng/ml subgroup than in the <1.5 ng/ml subgroups. When serum progesterone was elevated, the serum E2 levels and the number of retrieved oocytes were increased; however, fertilization rates, CPRs and IRs were significantly decreased.

In the day 5 ET group (Table 6), basal FSH levels, serum LH levels, fertilization rates and cleavage rates did not differ significantly between the two subgroups. The larger number of retrieved oocytes and higher serum E2 levels observed in the ≥1.75 ng/ml subgroup may indicate that this subgroup had a better ovarian response. However, this subgroup presented significantly lower IRs (29.5 % VS. 46.2 %, *p* = 0.002) and CPRs (31.3 % VS. 41.4 %, *p* < 0.001) compared with the <1.75 ng/ml subgroup with more transferred embryos. In addition, female age was significantly higher in the ≥1.75 ng/ml subgroup (31.5 ± 5.2 VS. 29.5 ± 4.4 y, *p* = 0.002).

## Discussion

This study showed that PE on the day of hCG administration decreased CPRs in both cleavage- and blastocyst-stage ET cycles using gonadotropin and GnRH agonist for controlled ovarian stimulation. In the day 3 ET cycles, a serum progesterone concentration of 1.0 ng/ml had a detrimental effect on CPRs. This detrimental effect worsened when the progesterone concentration reached 1.5 ng/ml. However, in the day 5 blastocyst-stage ET cycles, the detrimental effect did not appear until the progesterone concentration reached 1.75 ng/ml. The negative associations of PE with CPRs in both the day 3 and day 5 ET groups were the same after adjusting for confounders using multivariate analysis. Furthermore, the non-significant value of the test of the interaction between the developmental stage of the transferred embryos and PE with CPRs further confirmed that PE on the day of hCG administration was negatively associated with clinical pregnancy, regardless of the developmental stage of the transferred embryos.

Our results reaffirm the conclusions of previous retrospective studies that serum progesterone elevation on the day of hCG administration inversely impacts the pregnancy outcomes of patients undergoing IVF [1, 6, 10, 11]. In our study of more than 10,000 day 3 ET patients, we found that the detrimental effect of PE began at a threshold level 1.0 ng/ml and worsened when the level reached 1.5 ng/ml, which was similar to the findings of a recent meta-analysis [1] showing that progesterone levels (range: 0.8–1.1 ng/ml) have detrimental effects that worsen when the progesterone concentration reaches 1.2 ng/ml. Additionally, our study provides support for the nonlinear relationship between the progesterone levels on the day of hCG administration and pregnancy outcomes in fresh IVF cycles [1, 6].

Clinicians have utilized different strategies for controlling PE with the aim of avoiding its possible detrimental effects in fresh IVF cycles. Such PE control strategies include employing a mild stimulation protocol to achieve lower estradiol levels [19] or an earlier hCG trigger in high responders to avoid PE in the late follicular phase [20]. Other strategies that have been used to increase the probability of pregnancy in cases of PE in fresh IVF cycles included canceling fresh ETs and replacing the fresh embryos with frozen-thawed embryos in natural cycles. However, a recent retrospective analysis revealed that the incidence of PE (serum progesterone ≥1.57 ng/ml) in frozen-thawed embryo-transferred natural cycles was similar to that in fresh stimulated cycles [29].

Replacing cleavage-stage embryos with blastocyst-stage embryos prior to transfer has also been advocated since a previous study found that PE on the day of hCG administration decreased pregnancy rates in day 3 single ET cycles but not in day 5 single blastocyst transfer cycles. It was assumed that the endometrium had already significantly recovered from the impairment caused by supra-physiological steroid concentrations on the fifth luteal day [7, 21]. Although other studies have supported this hypothesis [9, 30], the reliability of this procedure still requires confirmation. In the present study, PE adversely affected CPRs when the progesterone level was ≥1.75 ng/ml in the day 5 blastocyst-stage ET group, a finding that is in agreement with previous suggestions that PE could impair IVF outcomes even with blastocyst transfers [8, 13, 22]. Furthermore, our interaction test did not reveal a link between the developmental stage of the transferred embryos and the effect of PE on clinical pregnancy [1].

In our study, we also used multivariate analysis to assess the detrimental effects of PE on CPRs [31] because parameters other than PE are closely related to IVF pregnancy outcomes [5, 32]. The results of this analysis showed that the number of top-quality transferred embryos had a positive effect on CPRs in both the day 3 and day 5 ET groups. Female age had a negative effect only in the day 5 ET group. In accordance with this result, female age is considered an independent factor related to clinical pregnancy because of age-related changes in the ovarian reserve, oocyte quality, embryo quality, and the uterine environment [33, 34]. However, oocyte number, which has been demonstrated to be correlated with PE [28] and strongly related to the probability of pregnancy and live birth [1, 3, 5, 6], was not confirmed to be related to CPRs in our study.

In IVF cycles, the luteal-phase endometrium is altered by the supra-physiological steroid concentration induced by ovarian hyperstimulation [21]. When the progesterone level is >1.5 ng/ml on the day of hCG administration, differences in gene expression in the endometrium occur after 36 h [35]. Our data also indicated that CPRs and implantation rates were obviously reduced when the progesterone level was >1.5 ng/ml in the day 3 ET group. A functional genomics analysis of the endometrium also confirmed that gene expression was altered in the endometrium as a result of PE on the day of hCG administration [16, 17]. In stimulated cycles, advanced endometrial maturation induced by PE may be related to the earlier closure of the implantation window and a reduction in pregnancy rates [14, 15]. Taken together, these observations indicate that endometrial receptivity is impaired by elevated progesterone, which may explain the poor IVF pregnancy outcomes. However, oocyte quality may not be affected [1, 18], as our data indicate that the fertilization and cleavage rates were not significantly different in the day 5 ET subgroups.

Comparisons between the subgroups of the day 3 and day 5 ET groups revealed that PE was associated with increased gonadotropin dose [1], oocyte number and E2 levels [5, 8, 11], which is in agreement with previous studies. These results may have occurred because each individual follicle contributes to the progesterone concentration in the circulation [36].

Interestingly, the cut-off levels for PE-induced impairment were different in the day 3 and day 5 ET groups in our study. Because the sample size of the day 3 ET group was sufficiently large (it contained more than 10,000 patients) and it included all patients regardless ovarian response [5, 6, 31], the cut-off levels (1.0 and 1.5 ng/ml) of the day 3 ET group may significantly represent the thresholds in the general population. However, the cut-off levels (1.75 ng/ml) of the day 5 ET group may be limited because of its smaller sample size (*n* = 1146). Moreover, the day 5 blastocyst-stage ET group tended to include good ovarian responders with high serum steroid levels as a result of the criteria for blastocyst transfer in our study.

The same progesterone assay used in our study and the quality control of the assay in our individual laboratory ensured the consistency of our progesterone measurement and minimized any variability in our assay [37]. However, because the assays in different institutes vary [3], our thresholds may differ from those of other studies that used different assays. Overall, our findings have the following clinical implications: serum progesterone levels should be controlled during controlled ovarian stimulation to avoid the detrimental effects of PE, replacing cleavage-stage embryos with blastocysts before transfer is not effective for avoiding the detrimental effects of PE, and utilizing a multivariate approach to assess the effects of PE in a given population and identifying specific cut-offs for that population in clinical practice is important.

In conclusion, this study shows that PE on the day of hCG administration is associated with decreased CPRs in GnRH agonist IVF/ICSI cycles regardless of the developmental stage of ET (cleavage versus blastocyst stage).

## Abbreviations

PE: Progesterone elevation
hCG: human Chorionic gonadotropin
IVF: In vitro fertilization
ICSI: Intracytoplasmic sperm injection
GnRH: Gonadotropin-releasing hormone
CPR: Clinical pregnancy rate
IR: Implantation rate
ET: Embryo transfer
FSH: Follicle-stimulating hormone
BMI: Body mass index
2PN: two Pronuclear
OR: Odds ratio
CI: Confidence interval
COS: Controlled ovarian stimulation
PCOS: Polycystic ovary syndrome
SD: Standard deviation
